# Supplementary material for: Learning to detect AI texts and learning the limits
Source: PLoS One. 2025 Oct 15;20(10):e0333007. doi: 10.1371/journal.pone.0333007 (PMC12527182; doi:10.1371/journal.pone.0333007)
Supplement: S2 File — All data and scripts used in the study, some additional descriptive statistics, scalable formate figures. (ZIP) [file pone.0333007.s002.tgz › data and scripts/OSF/scripts/analysis/Demographics/distribution_Demographics-age.pdf]

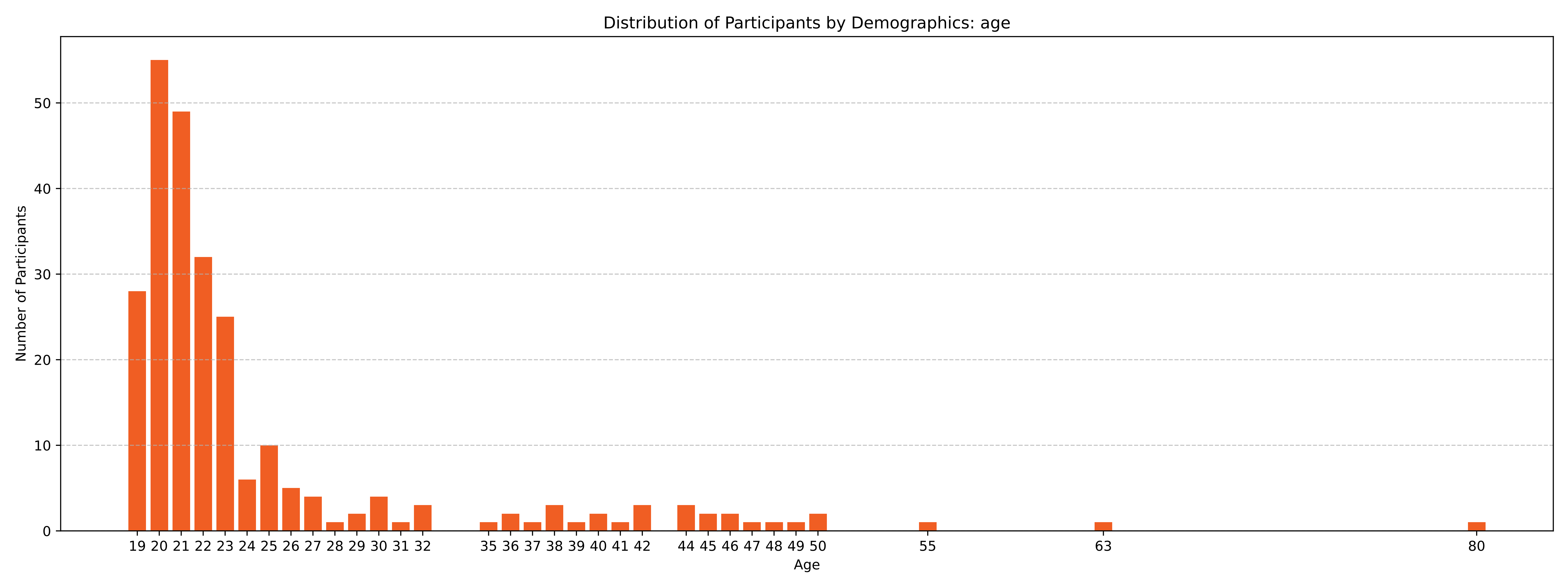

Distribution of Participants by Demographics: age
Number of Participants
0
10
20
30
40
50
19 20 21 22 23 24 25 26 27 28 29 30 31 32 35 36 37 38 39 40 41 42 44 45 46 47 48 49 50 55 63 80
Age
